# Spending time in the forest or the field: qualitative semi-structured interviews in a randomized controlled cross-over trial with highly sensitive persons

**DOI:** 10.3389/fpsyg.2023.1207627

**Published:** 2023-11-07

**Authors:** Katja Oomen-Welke, Tina Hilbich, Evelyn Schlachter, Alexander Müller, Andreas Anton, Roman Huber

**Affiliations:** ^1^Center for Complementary Medicine, Medical Center - University of Freiburg, Faculty of Medicine, University of Freiburg, Freiburg, Germany; ^2^Institute for Frontier Areas of Psychology and Mental Health (IGPP), Freiburg, Germany

**Keywords:** forest environment, therapeutic landscape, anxiety, depression, stress, relaxation, forest bathing, Shinrin-yoku

## Abstract

**Introduction:**

The effects of spending time in forests have been subject to investigations in various countries around the world. Qualitative comparisons have been rarely done so far.

**Methods:**

Sixteen healthy highly sensitive persons (SV12 score ≥ 18) aged between 18 and 70 years were randomly assigned to groups spending 1 h in the forest and in the field at intervals of one week. Semi-structured interviews were conducted after each intervention and analyzed using a mixed-methods approach of content analysis and grounded theory.

**Results:**

Both natural environments induced feelings of inner calmness, inner cleansing, joy, freedom, connectedness, strengthening qualities, and heightened body awareness. The forest environment additionally offered emotional shelter, and showed advantages in promoting inner strength and self-concentration.

**Discussion:**

People with previous negative experiences in the forest may feel safer in fields because of the wider view and better overview. Important preconditions are enough time and the absence of a judgmental authority. The two environments induced in part different but also similar emotions which might be useful to promote psychological well-being differentially.

## 1. Introduction

In the past 15 to 20 years, numerous studies in countries all over the world have investigated stays in forests and other natural environments for the purpose of health improvement (Kim et al., 2020; Andersen et al., 2021; Peterfalvi et al., 2021; Roviello et al., 2022). Spending time in forests seems to have positive effects on psychological well-being (Takayama et al., 2019; Stier-Jarmer et al., 2021), reduces anxiety, depression, and anger (Kotera et al., 2020), leads to reduced stress markers such as cortisol in saliva, adrenaline and noradrenaline in urine (Ochiai et al., 2015; Li et al., 2016; Dettweiler et al., 2017; Kondo et al., 2018; Wen et al., 2019; Kim et al., 2020), and lowers the systolic and diastolic blood pressure (Ideno et al., 2017). In questionnaires, participants reported a lower perception of stress (Oh et al., 2017; Stier-Jarmer et al., 2021), and a higher degree of relaxation (Timko Olson et al., 2020; Jones et al., 2021; Grabowska-Chenczke et al., 2022; Zwart and Ewert, 2022) compared to stays in a city environment or a hotel. After spending time in the forest, interviewees further reported less fatigue and increased vitality (Takayama et al., 2014; Furuyashiki et al., 2019; Bielinis et al., 2019a,b; Joung et al., 2020; Yau and Loke, 2020). So-called “Forest bathing” can decrease prefrontal cortex activity and induce relaxation (Park et al., 2007; Li, 2012).

Li et al. concluded that natural environments unfold their effects through sensory perceptions such as visual, acoustic, and olfactory sensations (Li, 2012). Therefore, in Japanese research, forest trips usually consist in perceiving the environment with all five senses, while standing, sitting, meditating, walking, hiking, or doing leisure activities. Some programs combine periods of activity and rest. So far, there is, however, no internationally uniform definition of forest therapy (Stier-Jarmer et al., 2021).

Another active factor are terpenes contained in essential oils from conifers. Terpenes reduce pro-inflammtory cytokines and modulate oxidative stress in *in vitro* experiments, animal models and experimental studies in humans (Oh et al., 2017; Wen et al., 2019; Andersen et al., 2021).

The sensory impressions in fields differ from those in forests. In a forest, the trees provide more shelter than plants in fields, which entails different light conditions. In forests, the sunlight filters through the canopy, resulting in a characteristic play of light and shadow. Fields usually lack protection from sun, wind or weather. On the other hand, they provide a wider view of the sky and the surroundings. Fields are usually divided into rectangular shapes which contain mainly one type of plant. Forests often provide more biodiversity than fields. In forests, natural processes lead to smells of resin or rotting wood.

Aron presented the concept of high sensitivity in 1997 (Aron and Aron, 1997). Highly sensitive persons (HSP) have a subtle perception that allows them to perceive stimuli like hunger, pain, medications, other people’s mood, and the media more intensively than other people. This may lead to more intense feelings and more emotional excitability (Vander Elst et al., 2019). Due to their low threshold of perception, they often feel overwhelmed by stimuli, which may lead to a prolonged reaction time (Aron et al., 2012). Therefore, they also suffer from stress more quickly than other people (Aron et al., 2012; Hinterberger et al., 2019). To protect themselves from overwhelming stimulation, they often tend to withdraw socially (Aron et al., 2012). This is why they often seem shy and introverted.

There are several questionnaires to measure sensitivity. The first was the Highly Sensitive Person Scale (HSPS) developed by Aron and Aron (1997) based on unidimensional theory of heterogenous sensitivities to internal and external stimuli. The original questionnaire consists of 27 questions to be rated from 0 to 7 (0 = “strongly disagree,” 7 = “strongly agree”) (Smolewska et al., 2006). There are various versions of this scale in different languages ranging from 10 (Iimura et al., 2023) to 27 items (Ershova et al., 2018; Chacón et al., 2021; Bordarie et al., 2022). Further research showed that high sensitivity is composed of the level of sensitivity and the level of processing ability, which both can independently vary in intensity (Ershova et al., 2018; Hinterberger et al., 2019). The Sensitivity and Processing Questionnaire (SV12) questionnaire takes this into account, which is why we chose this questionnaire to determine high sensitivity in our probands. Hinterberger et al. developed the long version of this questionnaire on a sample of 1,103 individuals and validated the short version SV12 by clinical use (Hinterberger et al., 2019).

We decided to invite HSPs to participate in our study because of their low threshold and strong reactions to subtle stimuli. We expected them to respond more clearly to the differences between forest and field. A recent research work showed a positive correlation between high sensitivity and connectedness to nature (Setti et al., 2022).

The research question was to what extent the subjective experiences of highly sensitive people in the forest and in the field differed qualitatively.

## 2. Methods

### 2.1. Participants

We included men and women aged between 18 and 70 years in our randomized controlled trial (RCT) using a cross-over design. To ensure a high level of sensitivity, we included individuals with SV12 (Hinterberger et al., 2019) total score (sum of items 1–6) > 18 points. Total scores of 16 to 18 are considered to be average. We excluded persons with mental disorders by using the ICD10 Symptom Rating Scale (ISR) (score > 1.7) and psychological interviews by a medical expert. Further exclusion criteria were serious concomitant physical or mental illnesses.

We recruited subjects between July and October 2020 through newspapers, public notices and Facebook posts and screened 150 persons using telephone interviews. Individuals who appeared to be suitable were screened again in a personal examination. From 43 individuals selected for the RCT, an independent researcher randomized 16 interviewees by using https://randomization.com/ (accessed on 07/08/2020, 21/08/2020, 02/10/2020, and 16/10/2020). One person dropped out after intervention 1 due to acute illness, and was replaced by a substitute. Only one of the interviewees was male.

### 2.2. Ethical approval

We registered the study in the European Clinical Trials Database (DRKS00020787) and applied Good Clinical Practice Guidelines (CPMP/ICH/135/95; Topic E6 (R1); and GCP-V), the Declaration of Helsinki and local laws. The local Ethics Committee reviewed and approved the protocol (EK-Freiburg registration number 70/20). All participants gave written informed consent before participation.

### 2.3. Environments

The study took place in South-West Germany. Subjects were recruited in a university city with 260.000 inhabitants (Freiburg i. Br.) surrounded by fields, forest and villages. The area has a population density of about 190 inhabitants per km^2^. We chose a mixed forest of deciduous and coniferous trees close to the inner city (10 to 20 min by car, bike or public transport) and a field in about the same distance. Fields in Germany are usually flat rectangles of land planted by one species of plant. We chose a field situated in a wide valley planted with corn. The Black Forest mountains were visible in the distance.

### 2.4. Intervention and control group protocol

This study is based on interviews with highly sensitive persons. The interviews were embedded in a cross-over RCT measuring the effects of forest and field environments on stress and psychological well-being. Interventions consisted in spending one hour in the forest or the field in small parallel groups of up to seven persons, respectively (Oomen-Welke et al., 2022).

Interviewees were selected randomly from the collective of the RCT (*n* = 43). To exclude weather bias, we conducted interventions simultaneously in a forest and a field site only a few kilometers distance from each other. We guided the groups on a short walk into the respective environments, where they spent 1 h meandering, sitting or standing, perceiving the surroundings. After 1 h, we conducted semi-narrative interviews using an interview guide (see 2.5 interview preparation). After a wash-out phase of one week, the groups met again at the respective other intervention site.

The study took place on eight Saturdays in August and October 2020. On each day we interviewed four individuals, two after each intervention.

### 2.5. Sample size

We planned two interviews from each group with a maximum of ten participants each (20%) of the quantitative study in order to achieve a representative group. As the groups were smaller (two to seven participants), we finally randomized eight times (from each parallel group) two participants, resulting in 32 interviews (16 interviewees out of 43 participants in eight groups, 37%). The 16 interviewees were chosen randomly by using an internet randomization list.^1^

They were informed (allocated) via email the evening before the intervention. None of the foreseen interviewees refused, one dropped out due to acute illness before the second intervention and was replaced by a randomly chosen substitute. Therefore, we finally counted 17 interviewees and 32 interviews.

### 2.6. Interviews

We created an interview guide as an orientation aid for the interviewers (Helfferich, 2011a). The first step was to collect as many questions as possible in an open brainstorming session. In a second step, the questions were checked for their suitability, collected into clusters and finally put into a structure for the interview guide. Guiding questions were as open and as broad as possible. We integrated supportive questions and specific follow-up questions into the guide in order to maintain the flow of the respondent’s speech (Helfferich, 2011a; Korstjens and Moser, 2017).

We chose “What were your perceptions and feelings during the stay?” as the main question with the guiding questions:

- How do you feel now?
- Is this perception specific to a forest/field environment?
- What kind of thoughts did you have during the intervention?
- What did you especially appreciate?
- What associations did you have?
- Miracle question: What would the ideal environment look like?

We trained all the interviewers (two medical students who both had previously trained and worked as nurses, a health care expert and a physician experienced in psychosomatics) to pay particular attention to neutral wording, long pauses, behavior encouraging more detailed descriptions and the avoidance of interpretation, evaluation, suggestions or the formulation of expectations. Interviews took place immediately after the interventions.

### 2.7. Evaluation strategy

We recorded the material with an audio-recorder, anonymized it by using participant IDs, transcribed and evaluated it in accordance with reconstructive interview analysis, a technique devised by Helfferich and Kruse (2007) and Kruse and Schmieder (2014). This is a mixed-methods approach of content analysis and grounded theory. In order to maintain the impartiality of the interviewers, we determined a detailed evaluation strategy (code definition, coding of interviews) after the interviews had been completed. The coders received training in qualitative research methods. Using MAXQDA software (Version 20.4.1), we extracted relevant themes from the interview data, grouped the statements under these theme headings and found codes to best describe the contents.

### 2.8. Translation

The interview was in German, which challenged us to find good translations into English for the publication. “Geborgenheit” is a German word that has no direct English equivalent. It describes a sensation that, for example, small children feel when cradled in a loving parent’s arms where no harm can reach them. It is a feeling of being sheltered physically and emotionally. For this article, we have used the word “emotional shelter.”

## 3. Results

The participants reported in both environments, but in different proportions, feelings of inner calmness, inner cleansing, strengthening, self-concentration, body awareness, freedom, and connectedness. Table 1 displays the number of interviews these qualities were mentioned in, Table 2 the group characteristics of the participants. Only in the forest did they mention emotional shelter.

**Table 1:** Qualities reported in the forest- and field environment. n = number of participants reporting the mentioned quality in the respective intervention, data from 32 interviews (16 in each intervention).

	Forest (*n*)	Field (*n*)
Inner calmness	12	9
Inner cleansing	12	4
Strengthening	10	3
Self-concentration	5	7
Body awareness	4	6
Freedom	4	4
Joy	2	5
Connectedness	3	5
Emotional shelter	13	0

**Table 2:** Group characteristics.

Group characteristics	Total
Participants total/both interventions (*n*)	17/15
Sex (f/m)	16/1
Age (years)	43 ± 16.6
SV12 total sensitivity	20.9 ± 1.5
ISR total score	0.36 ± 0.41
ISR symptom load (none/small/medium/heavy)	14/1/2/0
Employed (e/s/ue/rt)	7/5/1/3

f = female, m = male, e = employed, s = student, ue = unemployed, rt = retired; SV12 16–18 average sensitivity, max. score 24; ISR ICD-10 symptom rating total score (<0.5 no psychiatric disease).

### 3.1. Inner calmness

Inner calmness was an essential state that our participants sought in a natural environment. In order to find tranquility they said that they needed the feeling of having time available without deadline pressure and without having to do anything specific. Just having time by itself conveyed a feeling of relaxation. It helped them to let go.

“This immersion… When I hike, I get there after approximately 2 h, that it all stops in my head, this moving, what do I have to do? what do I want? what project am I working on..? Then I am here, yes, just here.” [20–08-29 P21 forest, lines 31–33, female, age 67, retired].

Guided stays with a scheduled time frame facilitate benefits from experiencing nature. Participants expressed difficulties in motivating themselves on their own to visit a natural environment and to allow themselves to do nothing but observe for one whole hour.

“It’s not about taking the time, it’s more about getting off one’s backside.” [20–08-08 P3 field, lines 77–78, female, age 22, student].

“For my standards, I looked at the insects for a surprisingly long time…” [20–08-08 P3 field; lines 26–29, female, age 22, student].

According to the participants, through tranquility they found “relaxation,” a “calmer pulse,” experienced their breathing “more consciously,” “calmer,” “deeper,” “freer” and perceived their chest as being “broader.” Together with their breathing their “thoughts stopped going round and round in circles.” “You seem to find a way back to yourself,” “to become more upright, more focused” and “more purposeful.” This “gives you strength.” Life is “no longer about doing,” but a “sense of joy in existence” arises. “Exercise” and “exertion,” as well as “leaning against a tree” or “lying down,” can promote “relaxation,” “letting go,” awareness of bodily sensations (“to sense oneself”) and, through this, inner clarity (“clearer in the head”) and “presence.”

Twelve participants reported experiencing calmness during the forest intervention, nine during the field intervention. The forest “radiates calmness and down-to-earthness” and thus has a “calming,” “relaxing,” “decelerating,” “grounding,” “thought-ordering,” “strengthening,” “invigorating,” and “inspiring” effect. The feeling of peace is conveyed through various sensory impressions. The typical, subdued lighting conditions, the play of light, shadow and sun have a “peaceful” effect. The sounds of civilization recede in favor of the sounds of the forest and water, greenery, the soft forest floor and the “special forest atmosphere.” The “primal nature of the forest,” as well as the feeling of “being at home,” “safe” and “emotionally sheltered,” convey feelings of “rootedness,” “grounding,” “inner centeredness and balance.” However, “it does not become too restrictive,” because you have freedom and space at the same time.

Time spent in a field also contributed to tranquility by facilitating a body sensation of being “relaxed,” “free,” “light,” “wide” and, “in a positive way, empty.” In contrast to the forest, the impression in a field seemed “too unlimited” to some people. They felt uncomfortable by “too much vastness” and “wind.” “Lacking protective cover,” they needed at least “high corn plants at their back” or an “elevated perch” to see who was coming.

The presence of natural water can further promote calmness and relaxation in both landscapes.

### 3.2. Inner cleansing

In our interviewees’ perceptions, there seemed to be several factors that contributed to a sense of “inner cleansing” in the forest, like “the greenness,” “the feeling of freshness” (fresh or aromatic smells associated with judging the air quality as “pure” and “healthy”), “cooler,” “moist” or “clear air,” which promoted a sense of “inner clarity.” This “purity” was associated with it being “good for your health.” It “rubbed off on people” and made them feel “clearer in their mind.”

“I think this sounds very strange, but I have the feeling that this greenness, freshness has rubbed off on me.” [20–08-15 P1 forest; lines 48–51, female, age 22, student].

The feeling of inner cleansing seems to relate particularly to the mental level. Twelve interviewees found that in the forest, and especially at viewpoints, they were able to “sort out their thoughts,” “free their mind” or “put their feelings in order” particularly well.

In the forest, what had previously felt “oppressive” was “cleared away.” What “weighed” them “down” could be “unloaded” in the forest. Then they reported “feeling light,” “carefree” and “coming out stronger” and “more confident.”

“When you realize you are mentally blocked or do not really know what to do, I find that you can recharge your batteries in the forest, really sort out your thoughts and then come out strengthened and stronger and confident.” [20–08-15 P3 forest; lines 54–56, female, age 22, student].

Only four participants reported a comparable effect from the field. Two interviewees pointed out that the forest environment had worked better to this end than the field environment.

### 3.3. Strengthening

In ten of the 16 forest interviews, the strengthening qualities of the forest were emphasized. Some individuals felt “more awake,” “refreshed,” “clearer,” “more alive,” “less tired,” “stronger,” “more active,” “happy” and “serene” after spending time in the forest. The forest experience with its “invigorating atmosphere” seemed to “inspire” more creativity and “energy” compared to the field experience. One participant attributed this to the “more varied” sensory input, others to cleansing mechanisms mentioned above.

“It’s just such a recharging of energy when you go into the forest, (…) like a superpower that just recharges you.. or like a big battery that recharges you. (..) Just the knowledge of how these trees, which are so firmly anchored in the ground and which are partly centuries old and simply go their way upwards and also interact with the other plants and trees. I feel that this whole network is heaving. It’s so, so big and if there is not any factor that’s kind of intimidating, which there just is not in the [German] forest, at least not during the day. >laughs< Then it gives you strength.” [20–08-15 P3 forest, lines 25–25, female, age 22, student].

With regard to the time spent in a field, the situation is not so clear. Only three interviewees felt invigorated. Two individuals reported that they had “become tired.” A sixth specified that her mind was awake now, but her body felt tired.

### 3.4. Self-concentration

By recharging one’s batteries, one becomes “more upright,” “more focused,” “more self-concentrated.” The factors that promote self-concentration that emerged from the interviews were “calmness,” “silence” (meaning fewer sounds of civilization in favor of sounds of the forest), “relaxation,” “color composition,” the “absence of distractions,” “time to sort out your thoughts,” “making sense of things,” focusing attention on body awareness (“to sense oneself”), not having to do anything, not having a schedule (“timelessness”), and the feeling of “being able to do what you want” without “the need of consideration,” without the need to “spare a thought to what other people think,” without having to meet others people’s expectations, without being subject to another’s judgment. The result is that one can gain distance from problems and concentrate on just being and a sense of freedom.

The effect of self-centering was greatly appreciated by our test persons. They described this state as feeling “calmer,” “more relaxed,” “more attentive,” “clearer,” “more present,” and “more at ease.” If one is guided by external influences, one can experience states of being overwhelmed. Self-centering in a natural environment can help people to regain their bearings (“get into the proper lane”) and thereby return to an inner harmony.

### 3.5. Body awareness

Participants reported that in a natural environment in general one can “gain distance from problems,” “sort out thoughts,” “relax” and “let go.” By letting go, one changes from “having to” and “doing” to “being,” “recognizes one’s own bodily sensations better” again, increases one’s body awareness (“to sense oneself”), which they experienced as a source of joy (“Actually these go together, joy and sensing oneself”). Achieving true being means “letting go of doubts,” reaching a state of “harmony” and “orienting oneself to one’s inner voice.” Factors that seem to draw attention to bodily sensations are rest, nature, haptic sensations (temperature, wind, cold), movement, exertion, “uneven ground” (in the forest through “stones and roots,” in the field through “corn stubble”). Consciously “feeling the ground” also leads to a “sense of connection with the earth.” “Leaning against a tree” or “lying on the ground” promote sensations of relaxation, which in turn strengthens the body sensation. Calmness and relaxation promote a bodily sensation of “opening up,” “broadening out” and “acquiring strength.”

The forest provided further favorable factors for both self-concentration and bodily awareness, among others “natural authenticity,” a feeling of “rootedness,” of “being grounded,” which is connected with “stability” and “safety” and a “more varied” and therefore “more stimulating” environment. The size and mightiness of the trees conveyed a change of scale.

“It’s a bit like looking into space. When you then realize how small you actually are and that the problems you see are simply in part exaggerated thoughts. So that in some cases one has too many thoughts and then one can concentrate again on oneself and reduce them. So that you really accept your life more again..” [20–08-15 P3 forest, lines 62–62, female, age 22, student].

### 3.6. Freedom

In each intervention, four participants uttered that they experienced a sense of freedom. Two participants felt it rather in the field environment because of its vastness. Two of them, on the other hand, felt freer in the forest because they felt “sheltered from the glances” and judgments of other people (“being hidden” and “sheltered” in the forest versus “being watched” and “being in a goldfish bowl” in the field). Two more participants experienced freedom in both the forest and field environment. One of them described that having “a lot of space” triggered a feeling of boundless freedom, which led to feelings of “liveliness,” “exuberance” and “lust for life.” One aspect of the feeling of freedom was not being judged (“I can do whatever I want here!”), as mentioned earlier.

In summary, one could “let go” and “relax.” They developed a sense of joy in existence (“It is no longer about doing, one may allow oneself just to be!”). As an aspect of joy, various participants named “curiosity,” “love of adventure,” the “joy of discovery,” “exploring and discovering mysteries.”

### 3.7. Joy

Our participants experienced joy in both forest and field. Joy as a reaction to the “beauty of nature” in terms of the overall impression, the colors (“joy comes with the colors”), “shapes” and forms, a particular flower, the green of the grass and plants, how a leaf turned as it fell, mushrooms, leaves, tree stumps, moss, rocks, stones, roots, the animals, the “lovely disorder,” the light (light in general, the light that fell through the trees as well as the play of light and shadow). “They have so amazingly many leaves, and yet it is so light, so joyfully light!” Some expressed joy in noticing changes in nature (e.g., seasonal changes, “joy in perceiving what’s different”). Many interviewees expressed pleasure in the “variety and diversity of nature.” Other aspects that evoked joy were the smells (fresh or aromatic, see “inner cleansing”), sounds (typical forest sounds, the “chirping of birds”), the “wide view,” the moving of one’s body, the feeling that their “soul” was “allowed to dangle.”

### 3.8. Connectedness

The participants identified feelings of connectedness (with the other participants, animals, trees, or nature as a whole) as factors that triggered joy. Connectedness arose from feeling a “sense of belonging” or “support,” among other things. Three participants mentioned feelings of connectedness in the forest, four in the field.

The participants mentioned the following aspects that fostered their feelings of connectedness:

- a sense of “security,”
- a certain “familiarity” (knowledge about nature, positive prior experiences of nature, especially childhood memories)
- “not having to think about anything”
- “to simply marvel, perceive, be a part of the big picture,” to be allowed to be completely absorbed in the moment, “then I am just here”
- “I can be completely who I am”
- “I can do what I want. I felt free.”
- the forest as a “place of refuge” for emotional pain like “grief or distress”
- haptic perception: touching the “white bark of a birch tree” which had “enchanted me when a child,” “to feel the softness of the ground”
- communication with plants and animals (talking to or hugging trees, being contacted by animals, feeling greeted or welcomed – “It was a great joy, amazement, that they [the ravens] paid me so much attention”)

### 3.9. Emotional shelter

Thirteen participants used terms of emotional shelter and reassurance in connection with their experiences in the forest (see “inner calmness”).

“I go into an organism that is one big living being that breathes, that welcomes me, you know, welcomes me, that makes me feel at home, that is authentic, alive, without any demands that I have to fulfill. I can completely be as I am. It is also a place of refuge, when I have to get rid of something like, let us say, grief or stress, but especially grief, the forest is very comforting for me.” [20–08-29 P21 forest; line 39, female, age 67, retired].

The “firm but soft,” “springy” forest floor (like leaning against a tree) conveys a feeling of “being supported” and “safe.” Together with the associations with the tall trees, which can maintain their “stability,” “safeness” and “uprightness” through a “good rooting in the ground,” the forest floor conveys a feeling of “roots.” The trees and undergrowth offer privacy and “protection from wind, rain, sun” (heat, UV radiation, glare), the “observation” of other people, “the noise of civilization” and give a feeling of “being surrounded, enclosed, protected, held in a positive way.”

“Such a joy, such a revival. It’s kind of like an envelope, simply around me. It’s such a joyful excitement…” [20–10-03 P37 field; lines 46–47, female, age 50, employed].

### 3.10. Stress factors

The participants named the following as stress factors in nature

- the weather (temperature, humidity),
- the light conditions (dense forest, darkness at night),
- monotonous landscapes,
- losing orientation (“not too dense … the feeling of knowing where I am,” “a forest can be threatening the moment I get lost,” knowing the way, the overview of the landscape and situation),
- the presence of animals (biting insects, larger mammals such as wild boars), possibly the sounds of animals, rustling that cannot be attributed, dogs barking,
- the presence and activity of other people in the forest (noise, the sounds of civilization, fear of being judged by other people, fear of being hurt (fast mountain bikers, “bad” people), too many people, people in too close proximity
- feelings of obligation (lack of time, deadline pressure)
- pollution of nature, damage to nature, disturbance of natural harmony (traffic noise, diseased trees, cleared areas, use of fertilizers and pesticides in agriculture).

### 3.11. Ideal natural environment

When asked about the ideal natural environment, most participants imagined a clearing or a meadow at the edge of the forest, to have on the one hand “the light, the brightness and the view into the distance,” and on the other hand the “emotional shelter,” the “protection of the trees.” One participant preferred the dim light that is typical of a forest. “Untouched nature,” “virgin forest,” “variation,” “biodiversity,” “vitality,” “a wide range of colors,” “mountains,” “water,” “animals,” and “insects” were mentioned as important factors.

„…a buzzing flower meadow, (…) like in the Alps. Then there is often real life in there, lots of different flowers and it just smells kind of delicious and it hums and buzzes, with trees around.” [20–08-29 P19 field; line 21, female, age 53, student].

Our test persons mentioned vitality or liveliness as an important feature of the ideal natural environment. They described liveliness as “colorfulness,” “great diversity,” in which “everything is allowed to grow next to each other.” Animals would “find refuge” there: insects, butterflies, mice, hedgehogs, squirrels. They associated diversity and liveliness with “togetherness,” with “community,” “full of joy.” Some compared these feelings with emotional states from childhood.

They described a “lively, harmonious togetherness” in the mixed forest as opposed to “mutual obstruction.” One had the feeling that “something lived or breathed there.” One could “linger there” for 2 h without doing anything, “simply marvel, perceive.” It “smelled pleasant,” was “green” and “alive.”

The forest was described as “imperfect” in a positive way, “the way nature wanted it to be,” a “lovely disorder,” “alive,” “strengthening/invigorating,” “a heaving network.”

In the field, interviewees expressed pleasure at seeing “a wide range of color.” A field gained liveliness by the “variety,” the “smells,” the “colors,” e.g., “multicolored flowers” and “butterflies” or a “buzzing meadow of flowers.” “Monocultures,” however, were judged “boring.” Our participants judged the field intervention site to be “pleasant” because “one could see the wooded mountains,” but also lifeless because of the “lack of crawling and flying insects.”

## 4. Discussion

This is, to our knowledge, the first qualitative study comparing experiences in different natural environments. Three qualitative studies about natural environments exist so far in medical research: One of them investigated the benefits of teenagers with psychiatric diseases and aggressive behavior watching the behavior of forest animals (Macháčková et al., 2021), the other one focused on the effects of outdoor adventure recreation (Zwart and Ewert, 2022), a third about experiences of veterans with post-traumatic stress injury (PTSI) in a forest therapy garden with mindfulness exercises, activities in nature, and therapeutic sessions (Poulsen et al., 2016). Qualitative studies allow a more detailed analysis of participants’ thoughts and emotions than questionnaires (Helfferich, 2011b), but are less robust and valid due to the small numbers of participants and an inherent possible selection bias. In order to assess possibly differential effects of natural environments on health and well-being, a qualitative approach offers the opportunity to collect a wide spectrum of impressions which might help further research to focus on relevant topics. By careful preparation of the interview guideline, the training of interviewers, standardized implementation of the interventions in parallel groups, the randomized selection of study participants from a larger cohort and structured data analysis, we fulfilled the state of the art criteria for qualitative studies (Kruse and Schmieder, 2014).

We compared two different natural environments (not a natural environment with a city) to bring out the similarities and the specifics of each. We described the polar properties of the forest and the field previously (Oomen-Welke et al., 2022).

A weakness of our study is a potential selection bias: Volunteers who answer a public call present us with the possibility of having a bias of an either very positive or very negative attitude toward the study aim. Many of our participants mentioned that they were very pleased to help to develop strategies that help highly sensitive persons, as they themselves had not experienced much support. None of them was critical.

Other weakness is the short duration of the intervention and that the study period extended over two different seasons, summer and autumn. On the other hand, the latter gave us the opportunity to study seasonal aspects of environmental perception. Furthermore, we do not know whether the benefits will also occur outside our guided setting. As most participants were female, our study is not representative for men.

We interviewed 15 participants after both interventions. One interviewee was ill at the time of the second intervention, and therefore was replaced by a substitute. Not all participants experienced both interventions under the same weather conditions, but as groups were in parallel in a cross-over design, the same number of participants experienced forest and field interventions at the same time.

Kondo et al. remarked that going outside often increases social contacts and physical activity, which may be mechanisms of stress reduction other than the perception of the natural environment (Kondo et al., 2018). We, therefore, asked participants not to interact with each other and limited the area of movement.

Our main finding is that field and forest in part cause the same emotions and in part cause different, possibly specific emotions. Thus, emotions experienced in both environments were inner calmness, inner cleansing, strengthening qualities, body awareness, freedom, and connectedness. Emotions caused mainly by the stay in the forest were emotional security and freedom in the sense of not being judged, because of the protection from observation provided by trees and undergrowth. In the field, participants uttered a different kind of freedom provided by the vastness, the wider view. Our participants reported many detailed, curious, emotional and imaginative expressions in the interviews, which fits well with the characterization of HSPs in the SV12 questionnaire (item 1, 3, 4, 6, 8, and 11) and corresponds to our intention to get distinct reactions to environmental stimuli.

These more or less specific emotions may be utilized therapeutically: individuals with a problem in trust, rootedness, or emotional shelter might, bringing our results to a new hypothesis, benefit from a stay in the forest but not from a stay in the field, while individuals with a freedom problem and difficulties with restricted space might benefit from stays in a large field.

The categories in our results, “inner calmness,’’ inner cleansing,” “strengthening,” “self-concentration,” “body awareness,” “freedom,” “connectedness,” and “emotional shelter” were codes we used for categories derived from our participants’ quotations. They are in part interrelated.

Researchers assumed that nature affects the human psyche through sensory impressions (Li, 2012; Stier-Jarmer et al., 2021). Based on our interviews, the sensory impressions could be divided into visual (light, colors, details), acoustic (polarity between natural and sounds and the sounds caused by civilization), olfactory, haptic, meteorological, and thermal perceptions. According to our participants, a wide variety of collaborating impressions seems to be essential.

Visually, colors and light conditions seem to have a major impact on mood and well-being. Bright and variegated colors seem to appeal to the sense of beauty, evoking joy and happiness. The beauty and diversity of natural flora and fauna seems to convey a sense that all is well and intact. The perceived inherent wisdom and intactness of nature together with the absence of obligations might have allowed our participants to relax. Nature connectedness might in turn benefit nature (Madan et al., 2019).

The connection between diversity and a sense of liveliness and cheerfulness stands in contrast to other research that did not find a connection to psychological benefits (Bratman et al., 2012).

Dark colors, on the other hand, seem to be stressful for some people. Variety and detail promote the joy of discovery and convey a sense of freedom. Acoustically, a polarity between natural and artificial sounds was evident from the interviews. The “silence” in the forest seems to be defined by the absence of man-made sounds in favor of the sounds of the forest. The majority seems to perceive the sounds of the forest as soothing. Cool, moist, fresh, good-smelling air seems to be associated with healthiness.

The feeling of emotional security and “being supported” seems to be created by the interplay of the soft forest floor, the presence of trees, smells, greenness, as well as the judgment of being enclosed in one’s own safe little world. The trees are perceived as peaceful, powerful living beings one can communicate with. They are role models in rootedness, steadfastness, uprightness, standing by yourself. Furthermore, they protect from the looks and thus the judgment of other people. For their ideal environment, many people want trees to lean against and provide shade. Emotional shelter was associated with secluded places, surrounded by trees – unless fear of people or animals arises.

Natural environments provided both connection and freedom. One can feel connected without facing any expectations or conditions.

“It’s difficult to describe. It’s just a good feeling. I do not have to deal with any problems, I can just do whatever I want. I felt free, connected to nature.” [20–08-22 P18 field; lines 12–16, male, age 25, employed].

Another important factor seems to be the absence of social judgement. Nature does not judge; one does not have to do anything in order to be allowed in. This might be another mechanism inducing a sense of emotional shelter.

“I’m fine here. I do not need to defend anything here. I do not need to explain myself here. (..) I can just be the way I am. I feel free and at ease.” [20–08-22 P21 field; line 31, female, age 67, retired].

Many people feel a compulsion to do something all the time. In nature, the participants reported being able to relax, let their soul dangle, feel free to do what they wanted or not to do anything. This might also be a consequence of the absence of judgement.

Having time to spend seems to play an important role. A stay in a natural environment can best unfold its effect if sufficient time is available. A too limited time frame can prevent the letting go that seems to be necessary for relaxation, the ordering of thoughts and feelings, anchoring in physicality, and self-centering.

The participants’ attitude influenced the effect of the environment. By redirecting attention from less to more beautiful aspects, or by breathing or being in a meditative state, one can reap more benefits from the environment.

“It’s a question of your attitude. To get the right thing for yourself out of what you have. (…) One can ignore the traffic if one manages to concentrate on breathing (..).” [20–08-29 P19 field; position 23–25, female, age 53, student].

Orientation seems to be an important prerequisite for a sense of security, which is essential for the calming effect of nature. Getting lost in the forest can cause anxiety. Orientation makes it possible to leave the forest or to go to certain places and thus serves one’s freedom of action. Secondly, an overview of the surroundings guarantees early recognition of dangers lurking in the background, which also provides security.

Participants pointed to conscious body awareness as an important mediator of the effect of the environment. Body awareness makes people conscious of tension and thus helps them to let go and relax. Conversely, relaxation seems to promote body awareness. Physical activity, exertion, shivering, the feeling of wind on the skin or the need to concentrate on the ground can also promote body awareness. Consciously feeling the ground beneath one’s feet can additionally provide a sense of connection to the earth. Body awareness in general seems to promote feelings of connectedness. Furthermore, body awareness promotes self-concentration. The participants apparently perceived self-connectedness as self-healing.

Guided stays get people to take more time than they would on their own. Many then succeed in letting go of obligations and immersing themselves in the atmosphere of the forest - arriving completely in the here and now. To this end, it seems beneficial to encourage silence and to hand over cell phones or switch them to airplane mode at the start. In this way, participants focus more on themselves and their surroundings. This promotes self-centering. Our participants mentioned that silence and turning off cell phones could minimize social pressure, allowing participants to focus more on themselves and their surroundings, which promotes self-concentration.

Knowing that one will be asked about it afterwards can promote a conscious experience. But it could also lead to people translating their sensations into language and diverting the focus from bodily sensation to rational comprehension.

In comparison with other research, our study confirms the restorative, calming, relaxing, and mood enhancing effects that have been reported in many publications (Oh et al., 2017; Takayama et al., 2019; Wen et al., 2019; Kotera et al., 2020; Stier-Jarmer et al., 2021). Poulsen et al.’s qualitative study (Poulsen et al., 2016) compares best with ours and shows many similarities. They found out that mindfulness exercises in nature and nature-based activities in combination with individual therapy sessions helped veterans with PTSI to become more attentive to body sensations and mental reactions. They experienced temperature, olfactory and visual impressions to impact their bodies directly. Our participants reported comparable effects by just perceiving the forest or field environment without exercises in mindfulness. Further research might determine whether the combination of nature based activities with exercises in mindfulness has additive effects.

The veterans searched for qualities in nature such as “placing no demands,” to be “accepted just the way you are.” This relates to the absence of social judgement our participants also appreciated nature. Other aspects that can be found in both studies are

- the preference some participants had of being shielded from people’s glances,
- the preference of others to overlook the whole area,
- the preference of leaning their backs against a tree or a pile of earth,
- to experience nature as calming,
- to consider old trees as symbols of stability,
- to find safety being surrounded by plants,
- to find relief in nature when the head is full of things to think about,
- the preference of wilderness to “arranged” nature.

Additionally, as this study ran for an extended period of time, veterans reported the effect of changes and rhythms in nature.

In the quantitative part of this study (Oomen-Welke et al., 2022), we found that forest stays improved feelings of security, integration and vitality. We also found that both natural environments increased vigor and reduced hostility, fatigue and depression/anxiety. All these findings are well in line with the qualitative results.

We believe that our work makes a valuable contribution to understanding how the previously observed effects of spending time in nature come about, how they are related, and what aspects can make nature therapy more effective. For further research, we suggest to verify and quantify our results by quantitative methods.

## 5. Conclusion

Our findings support the impressions of previous studies, that stays in a forest environment promote stress reduction and psychological well-being (Stier-Jarmer et al., 2021). In contrast to the stay in the field, the stay in the forest offered emotional shelter and hope. Both natural environments induced joy and connectedness.

We conclude that different environments result in different emotions and thoughts, which might differentially be usable for health promotion. We recommend further research to confirm and quantify our findings.

## Data availability statement

The raw data supporting the conclusions of this article will be made available by the authors, without undue reservation.

## Ethics statement

The studies involving humans were approved by Ethics Committee of Albert-Ludwigs-Universität Freiburg, Freiburg im Breisgau, Germany. The studies were conducted in accordance with the local legislation and institutional requirements. The participants provided their written informed consent to participate in this study. Written informed consent was obtained from the individual(s) for the publication of any potentially identifiable images or data included in this article.

## Author contributions

KO-W and RH: conceptualization, resources, supervision, and writing—review and editing. KO-W, RH, and AA: methodology. KO-W and TH: formal analysis. KO-W, TH, ES, and AM: investigation. KO-W: writing—original draft preparation. KO-W and ES: project administration. KO-W: funding acquisition. All authors have read and agreed to the published version of the manuscript.

## Footnotes

^1^ https://randomization.com/
